# Treatment of Psychological Symptoms in Patients with Cystic Fibrosis

**DOI:** 10.3390/jcm13195806

**Published:** 2024-09-28

**Authors:** Giovanna Campagna, Corrado Tagliati, Gian Marco Giuseppetti, Pietro Ripani

**Affiliations:** 1 UOSD CRR Fibrosi Cistica Ospedale “San Liberatore”, Dipartimento Materno Infantile, ASL Teramo, 64032 Atri, Italy; giovanna.campagna@aslteramo.it (G.C.); pietro.ripani@aslteramo.it (P.R.); 2 AST Ancona, Ospedale di Comunità, Via Marconi 1, 60040 Sassoferrato, Italy; 3 Medical Point Ancona, Via Trieste 21, 60124 Ancona, Italy; giuseppettigianmarco@gmail.com

**Keywords:** cystic fibrosis, treatment of psychological symptoms, cognitive–behavioral therapy, telemedicine, narrative medicine, counseling, family psychoeducation

## Abstract

The aim of this article is to identify and illustrate the most used psychological techniques in the field of cystic fibrosis (CF) and to help clinicians choose the most appropriate strategy among various possibilities. The disease and its medical treatments can be difficult to tolerate and can cause anxiety about health status or feelings of hopelessness and stress. The prevalence of depression and anxiety is 2.3 times higher in adults with CF than in community samples. A strong correlation has been identified between elevated psychological distress and unfavorable health outcomes, including, among others, impaired lung function, reduced BMI, an increased incidence of pulmonary exacerbations, and an elevated risk of transplantation. The use of psychological interventions is useful in addressing these common distresses in CF patients. Aware of the necessity of identifying efficacious interventions for all levels of depression and anxiety in CF patients, this study presents an overview of the research on psychological interventions for patients with CF, in order to complement the treatments suggested by the international guidelines on mental health in CF cases. In fact, the aim of this study is to conduct a review and quantitative synthesis of the psychological intervention techniques that are currently available for individuals with CF.

## 1. Introduction

Cystic fibrosis (CF) is a progressive, multisystem genetic disease, frequently characterized by respiratory distress due to recurrent pulmonary infections and associated damage [1]. Previously, the treatments available for CF were only capable of controlling symptoms and limiting complications.

Conventional therapeutic approaches were directed towards the treatment or prevention of damage to specific organ systems, employing a range of techniques, including the administration of nebulized medications for respiratory disorders, insulin for diabetes, and pancreatic enzyme supplementation.

Cystic fibrosis transmembrane conductance regulator (CFTR) modulators target specific genetic mutations, and have markedly transformed the lives of individuals with CF [2,3,4].

Over time, studies in CF management have led to a rapidly growing adult CF population, necessitating the development of new perspectives for patients (renewed parenting plans, adaptation to change) [5]. Nevertheless, the long-term consequences of these novel therapeutic modalities on the diverse manifestations of CF remain under investigation.

Although CF remains a life-shortening disease, survival rates have improved due to several factors. These include early diagnosis through routine neonatal screening, the combination of the CFTR modulators ivacaftor, tezacaftor, and elexacaftor [6], the promulgation of evidence-based guidelines to optimize nutritional and pulmonary health, and the development of interdisciplinary treatments specific to CF [7].

Notwithstanding the advancements, the quality of life for those living with CF remains significantly impaired by a considerable burden of painful symptoms and the necessity for daily treatments [8].

An epidemiological study of over 6000 individuals with CF revealed high rates of depression and anxiety, occurring at a frequency two to three times higher in those with CF than in the general population [9]. Moreover, the presence of untreated depression and anxiety in individuals with CF has been linked to adverse effects on health-related quality of life (HRQoL), adherence to treatment regimens, medical outcomes, and healthcare costs [9,10,11]. Therefore, international mental health guidelines, developed by the CF Foundation (CFF) and the European CF Society (ECFS), recommend routine screening, treatment, and preventive efforts for depression and anxiety as a component of standard CF care [12,13].

Psychological interventions have been demonstrated to be an efficacious approach for patients with CF, facilitating a reduction in emotional burden and the management of numerous mental health concerns. Moreover, a number of studies have evaluated the subjective and objective impacts of various psychological interventions on health outcomes in individuals with CF and their close family members. These studies have assessed a range of health-related outcomes, including quality of life and lung function. Nevertheless, no review has been conducted to synthesize the various techniques employed in the treatment of these patients.

The objective of this study is to conduct a review and quantitative synthesis of the psychological intervention techniques that are currently available for individuals with CF, including cognitive–behavioral therapy (CBT), telemedicine, narrative medicine, supportive counseling, and family psychoeducation, with the aim of improving psychological and psychosocial variables (e.g., quality of life, levels of stress or distress, psychopathology, etc.).

## 2. Cognitive–Behavioral Therapy Interventions

Access to evidence-based mental health care that meets the specific needs of individuals with CF is limited. Several factors contribute to this challenge, including difficulties related to insurance or financial support, a lack of understanding of CF by community workers, limited availability of mental health workers with the appropriate training, and long waiting lists [14].

A survey of 1454 CF caregivers concluded that additional training and educational resources are required for CF centers to implement mental health guidelines.

It is noteworthy that 47% of the healthcare professionals surveyed expressed a desire to receive training in cognitive behavioral therapy [15].

In the case of individuals with CF who present with mild symptoms of depression or anxiety, the guidelines suggest that monitoring, repeat screening, and the implementation of supportive interventions and psychoeducation should be considered.

For those with moderate to severe symptoms, the guidelines recommend the introduction of evidence-based interventions, such as cognitive–behavioral therapy (CBT), as a subsequent step [16].

CBT is an evidence-based intervention that helps CF patients develop adaptive coping skills.

It has been demonstrated to improve health-related quality of life (HRQoL) [17], promote emotional well-being, and treat depression and anxiety in populations with general and chronic illnesses [18,19].

The CFF/ECFS guidelines are recommended for individuals who have been screened for elevated symptoms of depression or anxiety [13].

Nevertheless, efficacious CBT and particular protocols are available for numerous medical conditions, including diabetes, COPD, HIV/AIDS, epilepsy, and cancer [20,21,22,23,24,25,26].

Recently, there have been notable advancements in the field of psychological interventions for individuals with CF.

These developments have been driven by the growing recognition of the urgent need for evidence-based CBT approaches in addressing the psychological challenges faced by this population.

A significant study was conducted with the objective of developing an innovative eight-session CBT-based intervention for the prevention and treatment of depression and anxiety, with the specific aim of tailoring the intervention to the needs of those with CF [27].

The results of these analyses indicate that a CF-specific CBT-based mental health intervention, designed to be integrated into routine CF care, is an acceptable approach.

A further study indicates that behavioral sleep interventions are efficacious in the treatment of common sleep difficulties in young people with CF. The results indicate that SLEEP-CF is an acceptable and feasible behavioral sleep intervention, even in a population with normal sleeping habits [28].

Based on the principles of CBT, a web-based psychological support program was developed for parents of children with CF who are severely distressed [29]. The web-based writing therapy comprises nine sessions, which have been adapted to meet the specific needs of caregivers. It was observed that, on average, the anxiety symptoms experienced by the caregivers, including fear of disease progression and symptoms of depression, decreased in a statistically significant and clinically relevant manner to a normal level between the pre-treatment and post-treatment periods. This resulted in a notable improvement in the quality of life of the caregivers, and the beneficial effects were sustained at the three-month follow-up assessment. The web-care method has demonstrated promise as an effective approach for enhancing the mental health and quality of life of parents. Another cognitive–behavioral counseling program has also been conducted for the benefit of caregivers of children, in which the semistructured interviews were subjected to thematic analysis [30]. In conclusion, there is some evidence that behavioral interventions targeting specific disease-related symptoms and behaviors are effective.

## 3. Telemedicine

Telemedicine, also known as ‘telehealth’ (or e-health), involves the use of electronic devices to exchange information for a variety of activities: ‘to keep patients and their healthcare providers in contact, especially those who live in remote geographical areas’ [31], ‘for the diagnosis and treatment of illness and injury, for research and evaluation, and for the continuing education of healthcare professionals’ [32]. The main goal of telemedicine is to improve the clinical health status of patients, for example, by helping to monitor respiratory parameters and improve access to care [33].

The World Health Organization (WHO) defines telemedicine as ‘the provision of health care services when patients and caregivers are separated by distance’.

Studies have shown that activating a telemedicine system in CF allows the patient to monitor certain biometric parameters such as oxygen saturation, weight, and respiratory function at home and send them directly to the referral center [31].

In the context of the global coronavirus (2019-nCoV) pandemic, which has resulted in significant limitations on access to medical environments, the role of telemedicine has been pivotal in maintaining contact with patients with CF who are experiencing psychological distress [34], and in a post-pandemic world, the necessity of this tool for psychological well-being should be taken into account.

A growing body of evidence suggests that e-health can effectively assist healthcare professionals in the management of individuals with CF. Consequently, telemedicine can facilitate clinical visits, adherence to daily treatment, including respiratory physiotherapy and exercise, the early identification of pulmonary exacerbations, and the management of psychological problems.

In response to the global coronavirus (2019-nCoV) pandemic, the CF team at the Bambino Gesù Children’s Hospital (Rome, Italy) devised a telehealth psychological support intervention for teenagers and young awCFs, as well as their caregivers. This intervention, which was made available during the period of social distancing, provided participants with cognitive–behavioral strategies to mitigate stress and emotional challenges associated with the pandemic [35]. The participants, comprising patients aged 12–36 years and caregivers of patients younger than 18 years, completed four individual telemedicine sessions with a psychologist. The sessions focused on self-care, coping skills, mood-enhancing exercises, and individual emotional challenges, as well as the use of screening instruments, including the Patient Health Questionnaire-8 item (PHQ-8) and the Generalized Anxiety Disorder-7 item (GAD-7). The intervention demonstrated a reduction in stress levels from the pre-test to the post-test period in both patients and parents. A high prevalence of depression and anxiety was reported at baseline in both groups (71%). A significant reduction in depressive symptoms was observed following the intervention (38%), whereas anxiety levels remained unchanged.

Another study examined the efficacy of administering Acceptance and Commitment Therapy (ACT) [36] in an electronic format in meeting patient needs and improving clinical symptoms [37]. ACT places an emphasis on the acceptance of circumstances, which serves to reduce the avoidance of anxiety and depressive symptoms commonly associated with CF. The participants were 28 awCFs with elevated clinical symptoms who completed six sessions of ACT for CF. The participants completed measures of depression, anxiety, and cognitive function at the outset of the study, following the intervention, and at the three-month follow-up. Lung function was assessed at three-month intervals prior to and following the administration of the treatment. The results of the study are as follows: the majority of participants (79%) opted for telehealth as their preferred mode of treatment. Ninety-six percent of participants completed all six sessions, and ninety-three percent expressed a strong desire to continue treatment with ACT. The results demonstrated a statistically significant reduction in a composite psychological distress score from before to after treatment in the ACT with FC group. Furthermore, the efficacy of ACT with FC delivered electronically was comparable to that of ACT delivered in person. Given the effect size associated with the reduction in psychosocial distress, it can be concluded that ACT with CF delivered via telemedicine or in person is a feasible and potentially effective treatment for improving anxiety and depressive symptoms

In another study, the Coping and Learning to Manage Stress with CF (CALM) intervention was developed for CF patients exhibiting high levels of depression or anxiety symptoms [38]. The CALM intervention is a telehealth cognitive–behavioral stress management (CBSM) intervention that has been specifically adapted for individuals with CF. CBSM is an established method for reducing symptoms of depression [39] and anxiety [40], improving quality of life [41], and increasing the use of coping skills and social support [42]. This pilot study is a randomized controlled trial (RCT) that compares awCFs who receive six telehealth-based CALM sessions. The participants completed the assessments at three time points: at baseline (week 0), following the intervention (week 8), and at the three-month follow-up (week 20). Eligible participants were required to be at least 18 years of age and to exhibit symptoms of depression or anxiety (i.e., scores ≥ 5 on the PHQ-9 or GAD-7). Following the administration of the six telehealth sessions, the CALM intervention was found to be an efficacious approach for the reduction of depressive and anxiety symptoms, as well as an improvement in coping strategies and health-related quality of life. Specifically, an e-health CBT intervention was developed, representing another digital mental health intervention for depression and anxiety in awCFs.

The eight-session program was led by a therapist and delivered via the Internet [43].

The e-health CF-CBT program provides psychoeducation and an introduction to fundamental CBT skills for the prevention and treatment of depression and anxiety, including relaxation techniques, behavioral activation, cognitive restructuring/adaptive thinking, and step-by-step exposure to cope with anxiety.

The program is designed to address the emotional challenges associated with living with CF, including CF-specific stressors (e.g., medical procedures, uncertainty about illness, hospitalizations, survivor guilt) and effective coping strategies [44].

The preliminary efficacy of the intervention is promising, as evidenced by statistically significant improvements in depression and anxiety, as well as in health-related quality of life, perceived stress, and adjustment.

The utilization of digital tools, such as mobile health (mHealth), has the potential to positively impact the physical and psychological well-being of adolescents with CF [45].

As defined by the WHO in 2011, mHealth is ‘the practice of medicine and public health supported by mobile devices, including but not limited to phones, patient monitoring devices, digital assistants, and other wireless devices’ [46]. The results of the study indicate that these new technologies have a beneficial impact on pediatric patients in two key areas: (a) enhanced enjoyment, socialization, and emotional expression; (b) diminished pain, anxiety, distress, and stress.

The implementation of telehealth and mHealth interventions for individuals with CF presents a valuable opportunity to overcome obstacles to mental health care access and reduce the overall healthcare burden.

## 4. Narrative Medicine

Narrative medicine can improve understanding of the discomfort and difficulties associated with the disease, thereby increasing patient motivation and participation in interdisciplinary care [47].

A fundamental aspect of this approach is the engagement in dialog with individuals who are afflicted with illness and pain.

Storytelling has become an effective way for patients to share their experiences of illness. It is regarded as an effective approach for gaining insight into how individuals make sense of their past experiences, situate themselves within the social context of their lives, and interpret and give meaning to the present. This process can also be applied to understanding and processing illness.

Narrative medicine interventions have the potential to be effective tools for facilitating behavior change and patient education [47]. By enabling patients to share their experiences with one another, these interventions can promote health and provide a sense of understanding.

The narrative medicine method has been applied by numerous researchers to patients with a variety of diseases.

In the case of Chagas disease, the use of this method provided sufficient and useful material for work on the disease context in biopsychosocial dimensions. This approach offers affected individuals the opportunity to raise their voices and enhance their life stories, thereby transforming themselves into the protagonists of their own narratives and knowledge [48].

Storytelling is a well-established and increasingly utilized research method in the fields of health and social sciences [49].

While used effectively in Chagas disease, narrative medicine is also being explored for patients with other pathologies and CF.

A study was conducted to identify the barriers and facilitators of physical activity in patients with a chronic disease using a storytelling methodology [50]. All patients who participated in this study had previously engaged in at least one physical activity promotion program, thereby establishing a foundation for the potential benefits of storytelling. The narrative therapy group intervention was employed with parents of children with physical health problems with the objective of facilitating a connection between parents and their abilities and resources. This technique provided parents with a secure environment in which to discuss difficulties, gain a new perspective on their situation, connect with other parents’ experiences, develop strategies for fostering independence in their children, and participate in challenging situations [51].

A qualitative narrative study was also conducted on patients on the waiting list for a kidney transplant who were hospitalized in a serious condition [52]. The life stories of patients with chronic kidney disease and its treatments reveal the discomfort associated with the condition and its treatment.

Moreover, another study has demonstrated that the narrative approach enhances the quality of care provided to children with chronic illnesses, including Crohn’s disease and HIV infection [53].

The use of narrative exploration has been demonstrated to be an effective method for understanding and addressing the needs of children with complex illnesses, and the use of this kind of approach enables the identification of the primary needs of patients with varying health conditions.

Another 2020 study also investigated the lived experiences of individuals with chronic illnesses in four patient groups: children with asthma, teenagers with diabetes, young adults with depression, and adult patients with chronic obstructive pulmonary disease (COPD) [54]. A narrative analysis was conducted to elucidate the manner in which the experiences of vulnerability were conveyed within the four patient groups, with the presentation of four discrete narratives, one from each patient group. The results demonstrate that the narratives elucidate the diverse competencies and capabilities that are requisite for navigating the experience of living with a chronic illness, contingent on the specific condition in question. Concurrently, the narratives illustrate how distinctive competencies and difficulties associated with chronic illness can be mitigated or perceived as assets. By drawing upon the resources of significant others, the surrounding environment, and the individual self, the storyteller can identify potential avenues for interpreting the experience of living with a chronic illness in a manner that may foster a sense of hope for the future.

In a separate study, disease narratives were employed to examine the experiences of individuals with chronic inflammatory bowel disease in the Netherlands. The findings indicate that these narratives facilitate the comprehension of the disease and the management of its consequences. They offer patients information and exemplars of effective coping strategies for their condition. Moreover, patients who read a positive illness narrative are more likely to internalize the emotional tone of the narrative and experience positive emotions such as joy, inspiration, and comfort [55].

Additionally, a narrative approach was employed, utilizing patients’ personal accounts to facilitate self-management of heart disease [56].

Other studies have likewise demonstrated the efficacy of narrative medicine in promoting disease self-management among individuals with type 2 diabetes [57,58]. The participants disseminated information and practical strategies for diabetes self-management, including the locations where healthier food alternatives could be purchased and the methods for cooking certain foods. Furthermore, they shared their experiences regarding various health services and professionals, as well as reliable resources for self-management. These processes may, in turn, contribute to the formation of a sense of community, which could facilitate peer support, empowerment, and active engagement in disease self-management.

Similarly, studies have been conducted in the CF field that explore these concepts, yet this area remains underexplored.

A 2023 study employed a narrative and play-based methodology to investigate the subjective experiences of young children (aged 3–6 years) with type 1 diabetes or CF [59].

The method combines visual tools, medical play, and narratives, thereby providing multiple avenues for children to express their perceptions and opinions and enabling the researcher to observe children’s internal experiences [60]. In this manner, the children grasped the essence of their condition through concrete experiences and the impact of the disease and treatment on their daily lives.

Another study assessed the efficacy of a novel therapeutic approach for CF, namely the metacognitive narrative imagination intervention. This approach integrates narrative and metacognitive mental imagery therapies [61]. The metacognitive theory posits that negative metacognitive beliefs are associated with mental disorders [62].

It is postulated that an anomalous cognitive model impairs the capacity to process ordinary emotional distress, thereby perpetuating it [63]. In metacognitive therapy, maladaptive metacognitive processes and beliefs are modified through positive mental imagery, thereby establishing more flexible thinking and reducing psychological distress [61]. A total of 13 patients, aged 10 to 17 years, received three one-hour sessions and were assessed for emotional functioning, anxiety, and depression at the outset of the study and at four and eight weeks thereafter. The participants reported significant improvements in anxiety and alterations in emotional functioning. Participants and their parents evaluated the metacognitive intervention with narrative imagery in terms of usability and preference.

Nevertheless, while the methodology and narrative medicine techniques employed in these studies are promising and effective, they still require further expansion.

## 5. Counseling

Mental health professionals face distinctive challenges when counseling patients with chronic diseases, such as CF.

It is therefore crucial for mental health professionals to possess the requisite skills, such as exemplary communication and active listening skills, to effectively engage with this specific age group.

Healthcare teams encounter distinctive challenges pertaining to the turbulence of adolescence and treatment compliance [64]. This necessitates a comprehensive understanding of the typical changes that occur during the course of child development. In recent years, scholars have conducted research on the experiences of adolescents with CF as they transition to adulthood. Their findings have highlighted differences in perspectives between male and female participants [65]. It is therefore recommended that professional counselors devote particular attention to this period of significant upheaval. Female participants reported experiencing depression, negative body image, and fear of diabetes as a potential complication of CF. In contrast, male participants demonstrated greater independence and only disclosed their fear of developing diabetes as the disease advanced.

It is recommended that professional counselors working with young people with CF consider these findings. Counselors should pay particular attention to issues related to treatment adherence, the potential for further physical complications of CF, and the importance of maintaining a positive outlook. It is therefore imperative that professional counselors working with young patients with CF tailor their interventions to specific developmental stages, exploring the level of acceptance of the disease and developing modalities that are appropriate for the age group in question.

It is of great importance that professional counselors prepare counseling interventions for young people with CF, taking into account the additional psychosocial and developmental challenges associated with the development. Many young patients share specific psychological and developmental needs as they grow up [66]. Nevertheless, young people with chronic illnesses face challenges in emotional development while managing physical symptoms [67].

Counseling professionals who demonstrate greater sensitivity to the progression and impact of this disease are better positioned to guide their intervention efforts. Professional counselors must have a comprehensive understanding of the disease and the various clinical complications that affect their patients in order to be sensitive to the trauma experienced by children and families coping with the disease [66ex64]. Furthermore, children with chronic illnesses may have an even higher incidence of mental health and psychosocial problems [68,69].

Consequently, professional counselors must enhance their understanding, competencies, and awareness of not only the physical challenges affecting children with CF, but also the emotional obstacles they confront.

For example, young people with CF may experience severe psychological difficulties (depression, hopelessness, suicidal ideation) and physical complications (impaired lung function, malnutrition) as their chronic condition progresses. The combination of these comorbidities contributes to the complexity of sustaining an intensive treatment modality [68,69,70] that also promotes psychosocial development and family system functioning [71]. It is the responsibility of professional counselors to delineate the circumstances under which parents will be informed of instances of self-harm or suicidal and homicidal ideation. Given the high prevalence of depression and suicidal ideation among children with chronic illnesses [70,71,72], it is evident that coping with the challenges and effects of CF is a constant concern for these children.

In order to provide evidence-based practices to clients with CF, as well as their loved ones, it is essential that professional counselors maintain an up-to-date knowledge base on mental health research related to this particular target population.

Although research indicates that individuals with chronic illnesses are at an elevated risk for developing depression [72], it is advised that depressive symptoms be regularly assessed and monitored, and that the level of illness acceptance in CF patients be examined.

It is essential that professional counselors working with children with CF further tailor their interventions to align with the specific symptom patterns exhibited by each patient. It is imperative that professional counselors establish a relationship with the child as an individual, rather than as a child with a disability [66].

In their 2004 study, Chesson et al. put forth recommendations for addressing the needs of children with chronic illnesses through counseling [73].

Firstly, it is essential that counselors ascertain the child’s understanding of the nature of counseling, including its purpose, potential benefits, the roles of the parties involved, and communication. While parental support and involvement are crucial aspects of counseling children [74,75], it is essential to avoid relying solely on parental reports as a substitute for exploring the child’s perspective on counseling. The impact of chronic illnesses such as CF on the family system is well documented [76]. However, within the therapeutic counseling relationship, children with CF must be empowered to perceive themselves as the experts in their own lives and mental health, regardless of their age.

Secondly, counselors working with children should limit the amount of talking they do in sessions. Instead, they should facilitate children’s involvement through natural modes of expression, such as play and drawing. It is recommended that counseling sessions be structured to include discussions alongside an activity, rather than relying solely on face-to-face conversation, in order to enhance the development of a therapeutic relationship. The formation of interdependent and healthy relationships with counselors may facilitate the development of independence and trust in children undergoing counseling, despite the challenges posed by disease progression [77]. The negative impact of chronic illness on social functioning [78] can result in social withdrawal, which can be exacerbated when counseling approaches are focused on the child utilizing adult techniques. It is therefore recommended that professional counselors implement age- and developmentally appropriate modifications to the counseling session. Similarly, the implementation of group counseling modalities should be considered as a means of counteracting the social isolation of these children.

Thirdly, professional counselors who develop a therapeutic relationship with a child with CF must include interactions that address the traumatic impact of living with the disease. Children with CF are subjected to physically stressful and painful experiences during medical treatment. The authors Geldard and Geldard proposed that professional counselors clarify the nature of counseling and differentiate it from medical treatment [74]. Furthermore, they propose the creation of an environment that is conducive to acceptance and the free expression of thoughts and feelings. It is suggested that professional counselors clarify the distinction between counseling and medical procedures (for example, that counseling does not involve the use of needles or painful medical procedures).

Similarly, Chesson et al. advise that counselors should endeavor to establish a therapeutic relationship with the child over the course of several shorter sessions, with the objective of fostering a sense of trust [73]. This approach avoids the potential pitfalls of rushing the process of building the counseling relationship and encourages active listening in order to gain a deeper understanding of the child’s world.

In the context of chronic illnesses such as CF, acute emotional reactions are an inevitable consequence, which may increase the child’s propensity to engage in self-injurious behavior [79]. It is essential that the counseling process be characterized by consistency and transparency with regard to the manner in which the child’s treatment progress is shared between adults.

It is of the highest importance that family members are involved in all aspects of the treatment of children with CF from the moment of diagnosis onwards. In fact, the diagnosis of a chronic illness has a significant impact on parents and families [70,71]. Nevertheless, family members (in particular, parents) frequently bear the responsibility of fostering an environment that enables children to cultivate resilience and autonomy. It is therefore recommended that professional consultants work with the family system to ensure that parents of children with CF have access to the necessary emotional support, family functioning and balance, including participation in one’s own counseling if necessary [80]. Similarly, professional counselors may be required to educate parents on the CF disease process, with a view to assisting them in identifying strategies to enhance resilience in their children [77]. Additionally, the counseling technique may be employed by members of the CF care team to facilitate the provision of education and support to patients regarding the initiation of CFTR modulator therapies (elexacaftor/tezacaftor/ivacaftor) [81].

## 6. Supportive Program for Family Caregivers

Family-centered care is considered optimal in pediatric healthcare because families play a crucial role in a child’s treatment [82,83]. This model is a healthcare paradigm that fosters collaboration between all parties involved [84,85], incorporates family members into the provision of care for children [84], and engages parents as co-decision-makers in their children’s healthcare when feasible [86].

The approach is biopsychosocial in nature, acknowledging the intricate interconnectivity between biological, psychological, and social elements that collectively shape an individual’s health and well-being [87].

The concept of family-centered care also extends to the child [88] and is associated with a number of positive outcomes. These include improved health and well-being of the child, increased parental satisfaction with the care provided, greater resources for improving health efficiency, increased access to care, and improved communication between families and transition providers [89]. Qualitative studies indicate that family-centered care offers a flexible framework for support that can be integrated into various sectors, including health, education, and social systems [90].

A number of studies have devised psychoeducational interventions for the families of children with illnesses.

The objective of one of the studies was to ascertain the impact of systemic family psychotherapeutic interventions on the quality of parent–child relationships and the optimization of the child’s glycemic control [91]. A significant reduction in glycated hemoglobin (HbA1c) values was observed following the implementation of a family psychotherapy plan.

Therefore, it can be concluded that systemic family psychotherapy is an effective method for managing the disease and strengthening parent–child relationships.

Another study was published a few years ago that examined parent education programs for families with children with special healthcare needs [92].

The presence of chronic health conditions in children and teenagers can result in a significant increase in the care burden experienced by parents [93].

Caregivers face increased burdens due to the need for home treatment and hospital visits [94]. The stress theory posits that the primary stressors experienced by caregivers include the level of care required, the severity of the illness, the patient’s health status, and behavioral issues. This can result in a subjective sense of burden, leading caregivers to feel overwhelmed and under pressure.

Consequently, there is a potential for caregivers to develop anxiety or depression, which may result in adverse mental and physical health outcomes [95]. Furthermore, it has been demonstrated that depressive symptoms in caregivers may have a detrimental impact on the patient’s ability to adhere to treatment regimens, which in turn may accelerate disease progression [96].

The advent of a chronic illness in one family member can have a profound impact on the entire family, affecting all family members [97].

Research indicates that a child’s illness can result in significant alterations to the family’s lifestyle. Parents of children with chronic illnesses frequently experience feelings of responsibility for their child’s condition, which can give rise to a range of emotional responses, including anxiety, guilt, helplessness, and feelings of incapacity [98].

Consequently, family members are compelled to redefine their roles, interaction patterns, and relationships within and outside the family in order to adapt to the new situation [99].

The coping strategies employed by parents of children with a disease such as CF may result in a redefinition of expectations and a re-evaluation of the importance of specific aspects of the child’s life. Such parents may experience a sense of loss, grieving the altered circumstances and the loss of what they previously considered to be their “normal” experience [97,98,99].

In addition to the emotional distress associated with grief and mourning, parents and children frequently confront uncertainty about the future. As regards existential distress, the most common existential needs of adults with CF are fears about the worsening of the disease, feelings about death and dying, fears about the worsening of cystic fibrosis, uncertainty about the future, and concerns for others [100].

This sense of uncertainty may be attributed to a lack of knowledge about the disease, its intricacies, treatment, potential side effects and management, the establishment of trusting relationships with healthcare providers, the quality of life, and the child’s capacity to cope.

This uncertainty is associated with elevated levels of emotional distress, diminished quality of life, and impaired psychosocial adjustment, as parents and children anticipate the worst and prepare for it [101]. This can impede parents’ capacity to perform fundamental tasks such as monitoring the child’s health, consistently enforcing behavioral expectations, promoting independence and self-management, and caring for and supervising other siblings [102].

It is evident that families frequently require assistance in a multitude of domains. One of the primary responsibilities of medical personnel is to provide support to patients.

In a study conducted in 2001, the development, implementation, and evaluation of a psychoeducational program for families with a child diagnosed with CF are described in detail [103]. To our knowledge, this is the only study so far that examined this type of program.

The impact of a family-oriented rehabilitation program in a recovery regimen on psychological symptoms reported by parents of chronically ill children is now well documented [104,105]. Indeed, one study examined the change in psychological symptoms and quality of life in children and adolescents with congenital heart disease, cancer, or CF after a family-oriented inpatient rehabilitation program [106]. Following rehabilitation, patients exhibited notable improvement in their symptoms, which was sustained for a period of six months in those who could be monitored.

Another study aimed to assess the efficacy of insight-oriented psychological therapy (IOT) as a treatment for unresolved grief (UG) in parents of children with CF compared to illness education [107]. IOT is designed to assist patients in developing new insights into their suffering, thereby facilitating a positive transformation in their internal world and state of mind.

In the context of potential UG in parents of children diagnosed with CF, IOT at the parental level may prove to be a valuable intervention for affected families.

The objective of the brief IOT was to assist parents in developing a more profound comprehension of the ramifications of UG pertaining to their child’s diagnosis on their daily coping mechanisms, interpersonal relationships, and apprehensions regarding their child’s future.

This was pursued with the intention of inciting a psychological transformation that would culminate in a sense of resolution in relation to their child’s CF diagnosis.

In conclusion, insight-oriented therapy appeared to facilitate the resolution of grief and bereavement in parents.

Subsequently, another study is being planned to develop and validate a support program for family caregivers of children with CF in Iran (2024–2025) [108].

Furthermore, the above studies demonstrated that reinforcing CF education about the disease, treatment, potential side effects, and quality of life enabled parents to identify solutions for psychological and disease management issues.

Providing parents with CF education immediately following diagnosis (during a period characterized by elevated stress, shock, and disbelief) may facilitate the acquisition and processing of information in a more accurate and comprehensive manner. The results indicate the necessity of addressing parents’ psychological distress and identifying a targeted intervention for parents, which may include psychoeducation and/or psychotherapy.

## 7. Other Techniques

In light of the documented impact of social support in other chronic diseases (e.g., diabetes, cancer, and heart disease) on mental health [109], physical health [110], and treatment adherence [111,112], one study investigated the influence of social support on awCFs [113]. The present study sought to elucidate the interrelationships between social support, mental and physical health, treatment activities, and disease-specific quality of life in a sample of awCFs. The results indicated that social support was associated with better emotional, social, and role functioning. It is possible that social support improves mental and physical health symptoms, leading to a greater ability to function in various areas of life. Social support also predicted better vitality, better body image, and better perception of overall health. Now that the link between social support and health-related quality of life in awCFs has been empirically explored, a multidisciplinary team can adopt this theory. Firstly, healthcare professionals can facilitate social engagement between patients and members of their communities. Secondly, they can design and implement modifications to clinical interventions that acknowledge the significance of social support on well-being. Furthermore, another study demonstrated that social support was associated with a reduction in the prevalence of mental and physical health symptoms among awCFs [113]. Indeed, a virtual one-to-one peer support program was established the following year for awCFs and their family members to assess the relationship between social support and health outcomes [114]. Social networks and peer support have been demonstrated to exert a beneficial influence on physical, mental, and social health outcomes through the enhancement of coping mechanisms, including problem-solving abilities, access to information, and perceived control. Such coping resources can serve as a buffer in periods of stress [115].

Peer support is a form of social support that involves the sharing of experiences and information among individuals with similar circumstances in a nonhierarchical relationship. It allows patients and family members to discuss complex health challenges that others in their social network may not understand. In one study [116], the objective was to develop a national CF-specific virtual peer support program to help people feel less alone and more supported. The objective was to address the needs of CF patients who sought to connect with their peers to exchange information regarding the management of daily life with a chronic disease. The most frequently reported benefits were social and emotional in nature, including feelings of increased support, decreased isolation, and the formation of new friendships. The data demonstrate that one-on-one virtual peer support is an acceptable and beneficial approach for both individuals with CF and their family members. It may therefore be replicable in clinical settings.

Another technique that has been demonstrated to be effective in addressing adherence issues in individuals with CF is motivational interviewing. In particular, the evidence substantiating the efficacy of motivational interviewing (MI) in enhancing adaptation and adherence in physical health and CF is examined. The review also provides an overview of the fundamental principles of MI, its application in medical settings, and recommendations for integrating MI techniques into the routine care of individuals with CF [117]. A pilot study implemented a journaling program with the objective of improving the mental and physical health outcomes of individuals with CF [118]. Eight adolescents between the ages of 12 and 17, all of whom had been diagnosed with CF and were under the care of a single CF clinical center, were sent weekly journal prompts via email. These prompts explored a range of topics, including treatment adherence, feelings of difference associated with CF, anxiety, depression, and interpersonal relationships. The participants expressed positive feedback regarding the study and that they experienced an improvement in their feelings of anxiety and depression. All participants indicated that they would recommend the study to others with CF. In conclusion, the journaling intervention for individuals with CF was found to be both feasible and well received.

Furthermore, there is a necessity to develop self-care programs as a psychological technique for well-being. A review of the literature was conducted to assess the efficacy of self-care support interventions for children and youth with asthma, CF, and diabetes [76]. The findings were synthesized narratively to examine the effectiveness of self-care support interventions on health status, psychosocial well-being, knowledge about the condition, use of health services, and participant satisfaction. There is compelling evidence that interventions targeting children and youth, utilizing telemedicine or group health methodologies, and delivered in community settings are highly effective. To exemplify, a study was conducted to ascertain the efficacy of an educational program on self-management, designated “Airways”, for children with CF between the ages of six and two and their caregivers with regard to adherence to aerosolized medications and airway clearance techniques (ACTs) [119]. Assessments were conducted at two time points: immediately prior to and following the intervention period, and at 6 and 12 months post-intervention. The pen-and-paper educational program was completed by the child and caregiver together at home on diary sheets over a 10-week period. The “Air Pathways” program provided information and behavioral exercises, developed practice assessment, treatment, and decision-making skills, and provided strategies to overcome barriers to treatment. The implementation of the program yielded positive outcomes with regard to adherence, self-management, children’s knowledge of ACT, and their attitudes towards the regular administration of both aerosol and ACT treatments. The favorable outcomes indicate that self-management is an efficacious educational instrument for school-aged children with CF and their caregivers.

Other methods may be employed as adjunctive therapy to improve quality of life. These include singing [120], which is considered a means of emotional expression, and yoga practice [121], which has been demonstrated to alleviate symptoms of pain, sleep disturbances, anxiety, and depression in CF patients.

## 8. Discussion

It is recommended that professional counselors employ multidimensional strategies when working with patients with special needs, as these individuals are at greater risk of psychological difficulties, including those with chronic illnesses and their parents.

Psychological interventions appear to offer a promising strategy for treating patients with chronic illnesses in general. In light of the multiple demands placed upon patients, it is recommended that personalized interventions be employed to enhance adaptation to the disease and adherence to therapy. Such interventions should increase CF knowledge and target coping skills. The primary objective was to identify and map the current evidence on the effectiveness of psychological interventions for CF.

This general overview of available techniques enables the selection of the most appropriate one for the patient, taking into account their needs, personality characteristics, specific problems, and the organizational context in which the work is carried out and the resources available.

It is imperative that measures are taken to ensure that CF centers have highly qualified professionals who are able to select the most appropriate strategies and personalized techniques to support the emotional burden of the disease.

There is still much to be discovered about the long-term effects of this condition.

It is therefore a priority to study a wider range of treatment modalities and digital interventions if clinical practice is to be improved.

Further studies may investigate additional techniques that have not yet been validated for use in CF but have demonstrated efficacy in other contexts. As an illustration, a promising technique for alleviating anxiety and depressive symptoms in CF patients is Rational–Emotional Behavior Intervention (REBT). This approach has demonstrated remarkable efficacy in the treatment of other diseases. REBT is one of the cognitive–behavioral therapies created by Ellis [122], which posits that all individuals are born with self-destructive tendencies. In the event of an action or situation that is contrary to their goals, values, or desires (typically a failure or rejection, for example), individuals have the option of experiencing positive emotions such as regret, disappointment, or frustration, which encourage them to revisit the situation and implement changes to address the adversity. Conversely, human beings have the capacity to choose whether to be overwhelmed by feelings of terror, panic, depression, self-pity, or self-doubt, which can impede their ability to adapt to adversity. Instead of their intrinsic goals and values, these emotions can influence their decision-making processes. REBT is an evidence-based therapeutic approach that aims to modify negative beliefs held by individuals about themselves, their future, and the world. As posited by REBT practitioners, irrational beliefs, including those characterized by fear, catastrophizing, demand, low frustration tolerance, and self/other/destructive cognitions, are identified as the primary causal factor in the development of emotional disorders in both healthy and ill individuals [123]. REBT employs a set of cognitive, emotional, and behavioral techniques to alter fundamental perspectives, which the therapist demonstrates and guides the patient in utilizing to significantly reduce distress [122]. This technique has been employed to alleviate the psychological distress of numerous patients afflicted with serious illnesses, including cancer [123], colorectal cancer [124], leukemia [125], congenital heart disease [126], visual impairment [127,128], type II diabetes [129], hypertension [130], Down syndrome and intellectual disability [131], post-traumatic stress disorder [132], autism [133], eating disorders [134], depression [135], and mental disorders in the elderly [136].

Other highly efficacious techniques for the treatment of anxiety and depression include mindfulness [137], which has yet to be explored in the context of CF, and psychodynamic therapy, which has the potential to enhance clinical remission [138], address somatoform pain disorders [139,140], and improve interpersonal functioning [141]. Further research is needed to expand the repertoire of techniques that can enhance the well-being of CF patients.

Future areas of research could include the psychological treatment effectiveness evaluation of CF patients and their caregivers, as well as further assessment of routine screening of multidisciplinary teams caring for CF patients.

A better understanding of the factors and mechanisms of mental distress could contribute to the development of advanced treatment programs that produce more optimal treatment outcomes.

The patient-centered recommendations could also be relevant for future interventions targeting psychosocial aspects.

## 9. Conclusions

Psychological interventions are reported to be efficacious in chronic diseases such as CF. Currently, early diagnosis and improved survival in CF emphasize the need for these interventions to help patients with coping mechanisms, adhere to prescribed therapies, and improve their quality of life.

In our opinion, a psychologist should always be included in the CF care team as their role is essential to reach these goals.

## Data Availability

Data are contained within the article.

## References

[1] Goss C.H. (2019). Acute pulmonary exacerbation in cystic fibrosis. Semin. Respir. Crit. Care Med..

[2] Clancy J.P., Cotton C.U., Donaldson S.H., Solomon G.M., Van Devanter D.R., Boyle M.P., Gentzsch M., Nick J.A., Illek B., Wallenburget J.C. (2019). CFTR modulator Theratyping: Current status, gaps and future directions. J. Cyst. Fibros..

[3] Jia S., Taylor-Cousar J.L. (2023). Cystic Fibrosis Modulator Therapies. Annu. Rev. Med..

[4] Taylor-Cousar J.L., Robinson P.D., Shteinberg M., Downey D.G. (2023). CFTR modulator therapy: Transforming the landscape of clinical care in cystic fibrosis. Lancet.

[5] Kazmerski T.M., West N.E., Jain R., Uluer A., Georgiopoulos A.M., Aitken M.L., Taylor-Cousar J.L. (2022). Family-building and parenting considerations for people with cystic fibrosis. Pediatr. Pulmonol..

[6] Purkayastha D., Agtarap K., Wong K., Pereira O., Co J., Pakhale S., Kanji S. (2023). Drug-drug interactions with CFTR modulator therapy in cystic fibrosis: Focus on Trikafta®/Kaftrio®. J. Cyst. Fibros..

[7] Dickinson K.M., Collaco J.M. (2021). Cystic Fibrosis. Pediatr. Rev..

[8] Bell S.C. (2020). The future of cystic fibrosis care: A global perspective. The future of cystic fibrosis care: A global perspective. Lancet Respir. Med..

[9] Quittner A.L., Abbott J., Hussain S., Ong T., Uluer A., Hempstead S., Lomas P., Smith B. (2020). Integration of mental health screening and treatment into cystic fibrosis clinics: Evaluation of initial implementation in 84 programs across the United States. Pediatr. Pulmonol..

[10] Smith B.A., Modi A.C., Quittner A.L., Wood B.L. (2010). Depressive symptoms in children with cystic fibrosis and parents and its effects on adherence to airway clearance. Pediatr. Pulmonol..

[11] Verkleij M., Maric M., Colland V., Nagelkerke A.F., Geenen R. (2017). Cognitive-behavioral therapy and eye movement desensitization and reprocessing in an adolescent with difficult-to control asthma. Pediatr. Allergy Immunol. Pulmonol..

[12] Abbott J., Havermans T., Jarvholm S., Landau E., Prins Y., Smrekar U., Staab D., Verity L., Verkleij M., ECFS Mental Health Working Group (2019). Mental Health screening in cystic fibrosis centres across Europe. J. Cyst. Fibros..

[13] Quittner A.L., Abbott J., Georgiopoulos A.M., Goldbeck L., Smith B., Hempstead S.E., Marshall B., Sabadosa K.A., Elborn S., International Committee on Mental Health (2016). International Committee on Mental Health in Cystic Fibrosis: Cystic Fibrosis Foundation and European Cystic Fibrosis Society consensus statements for screening and treating depression and anxiety. Thorax.

[14] Mueller A.E., Georgiopoulos A.M., Reno K.L., Roach C.M., Kvam C.M., Quittner A.L., Lomas P., Smith B.A., Filigno S.S. (2020). Introduction to cystic fibrosis for mental health care coordinators and providers: Collaborating to promote wellness. Health Soc. Work.

[15] Abbott J., Elborn J.S., Georgiopoulos A.M., Goldbeck L., Marshall B.C., Sabadosa K.A., Smith B.A., Quittner A.L. (2015). Cystic Fibrosis Foundation and European cystic fibrosis society survey of cystic fibrosis mental health care delivery. J. Cyst. Fibros..

[16] Friedman D., Smith B.A., Bruce A., Schwartz C.E., Lee H., Pinsky H., Gootkind E., Hardcastle M., Shea N., Roach C.M. (2022). Feasibility and acceptability of a CF-specific cognitive-behavioral preventive intervention for adults integrated into team-based care. Pediatr. Pulmonol..

[17] Getu M.A., Chen C., Panpan W., Mboineki J.F., Dhakal K., Du R. (2021). The effect of cognitive behavioral therapy on the quality of life of breast cancer patients: A systematic review and meta-analysis of randomized controlled trials. Qual. Life Res..

[18] Butler A.C., Chapman J.E., Forman E.M., Beck A.T. (2006). The empirical status of cognitive-behavioral therapy: A review of meta-analyses. Clin. Psychol. Rev..

[19] Fordham B., Sugavanam T., Edwards K., Stallard P., Howard R., das Nair R., Copsey B., Lee H., Howick J., Hemming K. (2021). The evidence for cognitive behavioural therapy in any condition, population or context: A meta-review of systematic reviews and panoramic meta-analysis. Psychol. Med..

[20] Antoni M.H., Lechner S.C., Kazi A., Wimberly S.R., Sifre T., Urcuyo K.R., Phillips K., Glück S., Carver C.S. (2006). How stress management improves quality of life after treatment for breast cancer. J. Consult. Clin. Psychol..

[21] Antoni M.H., Lechner S., Diaz A., Vargas S., Holley H., Phillips K., McGregor B., Carver C.S., Blomberg B. (2009). Cognitive behavioral stress management effects on psychosocial and physiological adaptation in women undergoing treatment for breast cancer. Brain Behav. Immun..

[22] Cully J.A., Stanley M.A., Deswal A., Hanania N.A., Phillips L.L., Kunik M.E. (2010). Cognitive-behavioral therapy for chronic cardiopulmonary conditions: Preliminary outcomes from an open trial. Prim. Care Companion J. Clin. Psychiatry.

[23] Laperriere A., Ironson G.H., Antoni M.H., Pomm H., Jones D., Ishii M., Lydston D., Lawrence P., Grossman A., Brondolo E. (2005). Decreased depression up to one year following CBSM+ intervention in depressed women with AIDS: The Smart/EST women’s project. J. Health Psychol..

[24] Thompson N.J., Walker E.R., Obolensky N., Winning A., Barmon C., Diiorio C., Compton M.T. (2010). Distance delivery of mindfulnesss-based cognitive therapy for depression: Project UPLIFT. Epilepsy Behav..

[25] Safren S.A., O’Cleirigh C., Tan J.Y., Raminani S.R., Reilly L.C., Otto M.W., Mayer K.H. (2009). A randomized controlled trial of cognitive behavioral therapy for adherence and depression (CBT-AD) in HIV-infected individuals. Health Psychol..

[26] Soo H., Lam S. (2009). Stress management training in diabetes mellitus. J. Health Psychol..

[27] Friedman D., Kaskas M.M., Quittner A.L., Smith B.A., Georgiopoulos A.M. (2022). Patient engagement in the development of CF-CBT: A cystic fibrosis-specific cognitive-behavioral intervention for adults. Front. Psychol..

[28] Canter K.S., Strang A., Wilks S. (2022). Acceptability and feasibility of a brief behavioral sleep intervention for youth with CF. J. Cyst. Fibros..

[29] Fidika A., Herle M., Lehmann C., Weiss C., Knaevelsrud C., Goldbeck L. (2015). A web-based psychological support program for caregivers of children with cystic fibrosis: A pilot study. Health Qual. Life Outcomes.

[30] Moola F.J., Henry L.A., Huynh E., Stacey J.A., Faulkner G.E. (2016). They know it’s safe—They know what to expect from that face: Perceptions towards a cognitive-behavioural counselling programme among caregivers of children with cystic fibrosis. J. Clin. Nurs..

[31] Esposito S., Rosafio C., Antodaro F., Argentiero A., Bassi M., Becherucci P., Bonsanto F., Cagliero A., Cannata G., Capello F. (2023). Use of Telemedicine Healthcare Systems in Children and Adolescents with Chronic Disease or in Transition Stages of Life: Consensus Document of the Italian Society of Telemedicine (SIT), of the Italian Society of Preventive and Social Pediatrics (SIPPS), of the Italian Society of Pediatric Primary Care (SICuPP), of the Italian Federation of Pediatric Doctors (FIMP) and of the Syndicate of Family Pediatrician Doctors (SIMPeF). J. Pers. Med..

[32] World Health Organization (2022). International Telecommunication Union WHO-ITU Global Standard for Accessibility of Telehealth Services.

[33] Burke B.L., Hall R.W. (2015). Telemedicine: Pediatric Applications. Pediatrics.

[34] Fainardi V., Capoferri G., Tornesello M., Pisi G., Esposito S. (2023). Telemedicine and Its Application in Cystic Fibrosis. J. Pers. Med..

[35] Graziano S., Boldrini F., Righelli D., Milo F., Lucidi V., Quittner A., Tabarini P. (2021). Psychological interventions during COVID pandemic: Telehealth for individuals with cystic fibrosis and caregivers. Pediatr Pulmonol..

[36] Hayes S.C. (2004). Acceptance and commitment therapy, relational frame theory, and the third wave of behavioral and cognitive therapies. Behav. Ther..

[37] O’Hayer C.V., O’Loughlin C.M., Nurse C.N., Smith P.J., Stephen M.J. (2021). ACT with CF: A telehealth and in-person feasibility study to address anxiety and depressive symptoms among people with cystic fibrosis. J. Cyst. Fibros..

[38] Bathgate C.J., Kilbourn K.M., Murphy N.H., Wamboldt F.S., Holm K.E. (2022). Pilot RCT of a telehealth intervention to reduce symptoms of depression and anxiety in adults with cystic fibrosis. J. Cyst. Fibros..

[39] Antoni M.H., Lehman J.M., Kilbourn K.M., Boyers A.E., Culver J.L., Alferi S.M., Yount S.E., McGregor B.A., Arena P.L., Harris S.D. (2001). Cognitive-behavioral stress management intervention decreases the prevalence of depression and enhances benefit finding among women under treatment for early-stage breast cancer. Health Psychol..

[40] Antoni M.H., Cruess D.G., Cruess S., Lutgendorf S., Kumar M., Ironson G., Klimas N., Fletcher M.A., Schneiderman N. (2000). Cognitive-behavioral stress management intervention effects on anxiety, 24-hr urinary norepinephrine output, and T-cytotoxic/suppressor cells over time among symptomatic HIV-infected gay men. J. Consult. Clin. Psychol..

[41] Penedo F.J., Traeger L., Dahn J., Molton I., Gonzalez J.S., Schneiderman N., Antoni M.H. (2007). Cognitive behavioral stress management intervention improves quality of life in Spanish monolingual hispanic men treated for localized prostate cancer: Results of a randomized controlled trial. Int. J. Behav. Med..

[42] Lutgendorf S.K., Antoni M.H., Ironson G., Starr K., Costello N., Zuckerman M., Klimas N., Fletcher M.A., Schneiderman N. (1998). Changes in cognitive coping skills and social support during cognitive behavioral stress management intervention and distress outcomes in symptomatic human immunodeficiency virus (HIV)-seropositive gay men. Psychosom. Med..

[43] Verkleij M., Georgiopoulos A.M., Barendrecht H., Friedman D. (2023). Pilot of a therapist-guided digital mental health intervention (eHealth CF-CBT) for adults with cystic fibrosis. Pediatr. Pulmonol..

[44] Verkleij M., Georgiopoulos A.M., Friedman D. (2021). Development and evaluation of an internet-based cognitive behavioral therapy intervention for anxiety and depression in adults with cystic fibrosis (eHealth CF-CBT): An international collaboration. Internet Interv..

[45] Valero-Moreno S., Lacomba-Trejo L., Montoya-Castilla I., Pérez-Marín M. (2021). Is mHealth a useful therapy for improving physical or emotional health in adolescents with cystic fibrosis? A systematic review. Curr. Psychol..

[46] World Health Organization (2011). MHealth. New Horizons for Health through Mobile Technologies.

[47] De Sire A., Marotta N., Drago Ferrante V., Calafiore D., Ammendolia A. (2023). Effects of multidisciplinary rehabilitation in a patient with Ehlers-Danlos and Behçet’s syndromes: A paradigmatic case report according to the narrative medicine. Disabil. Rehabil..

[48] Pereira-Silva F.S.A., Braga Corrêa de Mello M.L., Cremonini de Araújo-Jorge T. (2022). Chagas disease: Tackling the invisibility through the analysis of life histories of chronic patients. Cien. Saude Colet..

[49] McCall B., Shallcross L., Wilson M., Fuller C., Hayward A. (2021). Storytelling as a Research Tool Used to Explore Insights and as an Intervention in Public Health: A Systematic Narrative Review. Int. J. Public Health.

[50] de Boer J.J., Feleus A., Hesselink A., Siemonsma P., Verhoef J., Schmitt M. (2022). Using storytelling methodology to identify barriers and facilitators of sustained physical activity in patients with a chronic disease: A qualitative study. BMJ Open.

[51] Haselhurst J., Moss K., Rust S., Oliver J., Hughes R., McGrath C., Reed D., Ferguson L., Murray J. (2021). A narrative-informed evaluation of tree of life for parents of children with physical health conditions. Clin. Child Psychol. Psychiatry.

[52] Naro M., Plotton C., Vassal P., Xavier G. (2020). Narrative inquiries from kidney transplant patients: From the onset of the disease to the transplant. Nephrol. Ther..

[53] Continisio G.I., Nunziata F., Coppola C., Bruzzese D., Spagnuolo M.I., Guarino A. (2021). Enhancing the care of children with chronic diseases through the narratives of patient, physician, nurse and carer. Scand. J. Psychol..

[54] Synnes O., Orøy A.J., Råheim M., Bachmann L., Ekra E.M.R., Gjengedal E., Høie M., Jørgensen E., Michaelsen R.K.A., Sundal H. (2020). Finding ways to carry on: Stories of vulnerability in chronic illness. Int. J. Qual. Stud. Health Well-Being.

[55] Brokerhof I.M., Ybema J.F., Bal P.M. (2020). Illness narratives and chronic patients’ sustainable employability: The impact of positive work stories. PLoS ONE.

[56] Sentell T., Kennedy F., Seto T., Vawer M., Chiriboga G., Valdez C., Garrett L.M., Paloma D., Taira D. (2019). Sharing the Patient Experience: A “Talk Story” Intervention for Heart Failure Management in Native Hawaiians. J. Patient Exp..

[57] Gucciardi E., Reynolds E., Karam G., Beanlands H., Sidani S., Espin S. (2021). Group-based storytelling in disease self-management among people with diabetes. Chronic Illn..

[58] Lohr A.M., Vickery K.D., Hernandez V., Ford B.R., Gonzalez C., Kavistan S., Patten C.A., Njeru J.W., Novotny P.J., Larkey L.K. (2023). Stories for change protocol: A randomized controlled trial of a digital storytelling intervention for Hispanic/Latino individuals with type 2 diabetes. Contemp. Clin. Trials.

[59] DeCosta P., Skinner T.C., Sørensen J.L., Topperzer M.K., Grabowski D. (2023). Young children’s perspectives on treatment and care: A qualitative study using narrative and play-based interviewing. J. Pediatr. Nurs..

[60] Kelly K.R., Bailey A.L. (2020). Narrative story stem methodologies: Use and utility of quantitative and qualitative approaches across the lifespan. Narrat. Inq..

[61] Russell J.K., Strodl E., Connolly J., Kavanagh D.J. (2021). A Metacognitive Intervention of Narrative Imagery for young people with cystic fibrosis: A feasibility study. J. Health Psychol..

[62] Wells A. (2009). Metacognitive Therapy for Anxiety and Depression.

[63] Gebhardt P., Caldarone F., Westhoff-Bleck M., Olsson K.M., Hoeper M.M., Park D.H., Stapel B., Breitner M.H., Werth O., Heitland I. (2022). Metacognitive short-term intervention in patients with mental disorders following cardiovascular events. Front. Psychiatry.

[64] Withers A.L. (2012). Management issues for adolescents with cystic fibrosis. Pulm. Med..

[65] Berge J.M., Patterson J.M., Goetz D., Milla C. (2007). Gender differences in young adults’ perceptions of living with cystic fibrosis during the transition to adulthood: A qualitative investigation. Fam. Syst. Health.

[66] Thompson C.L., Henderson D.A. (2007). Counseling Children.

[67] Dahlbeck D.T., Lightsey O.R. (2008). Generalized self-efficacy, coping, and self-esteem as predictors of psychological adjustment among children with disabilities or chronic illnesses. Child. Health Care.

[68] Canning E.H., Haner S.B., Shade K.A., Boyce W.T. (1992). Mental disorders in chronically ill children: Parent-child discrepancy and physician identification. Pediatrics.

[69] Barnes A.J., Eisenberg M.E., Resnick M.D. (2010). Suicide and self-injury among children and youth with chronic health conditions. Pediatrics.

[70] Anderson D.L., Flume P.A., Hardy K.K. (2001). Psychological functioning of adults with cystic fibrosis. Chest.

[71] O’Haver J., Moore I.M., Insel K.C., Reed P.G., Melnyk B.M., Lavoie M. (2010). Parental perceptions of risk and protective factors associated with the adaptation of siblings of children with cystic fibrosis. Pediatr. Nurs..

[72] Quittner A.L., Barker D.H., Snell C., Grimley M.E., Marciel K., Cruz I. (2008). Prevalence and impact of depression in cystic fibrosis. Curr. Opin. Pulm. Med..

[73] Chesson R.A., Chisholm D., Zaw W. (2004). Counseling children with chronic physical illness. Patient Educ. Couns..

[74] Geldard K., Geldard D. (2008). Counselling Children: A Practical Introduction.

[75] Morison J.E., Bromfield L.M., Cameron H.J. (2003). A therapeutic model for supporting families of children with a chronic illness or disability. Child. Adolesc. Ment. Health.

[76] Kirk S., Beatty S., Callery P., Gellatly J., Milnes L., Pryjmachuk S. (2013). The effectiveness of self-care support interventions for children and young people with long-term conditions: A systematic review. Child. Care Health Dev..

[77] Juntunen C.L., Atkinson D.R. (2002). Counseling across the Lifespan: Prevention and Treatment.

[78] Last B.F., Stam H., Onland-van Nieuwenhuizen A.M., Grootenhuis M.A. (2007). Positive effects of a psychoeducational group intervention for children with a chronic disease: First results. Patient Educ. Couns..

[79] Storlie C.A., Baltrinic E.R. (2015). Counseling Children With Cystic Fibrosis: Recommendations for Practice and Counselor Self-Care. Prof. Couns..

[80] Tluczek A., Laxova A., Grieve A., Heun A., Brown R.L., Rock M.J., Gershan W.M., Farrell P.M. (2014). Long-term follow-up of cystic fibrosis newborn screening: Psychosocial functioning of adolescents and young adults. J. Cyst. Fibros..

[81] Landess L., Prieur M.G., Brown A.R., Dellon E.P. (2024). Exploring perceptions of and decision-making about CFTR modulators. Pediatr. Pulmonol..

[82] Carrington L., Hale L., Freeman C., Qureshi A., Perry M. (2021). Family-Centred Care for Children with Biopsychosocial Support Needs: A Scoping Review. Disabilities.

[83] Arakelyan S., Maciver D., Rush R., O’hare A., Forsyth K. (2019). Family factors associated with participation of children with disabilities: A systematic review. Dev. Med. Child. Neurol..

[84] Kuo D.Z., Houtrow A.J., Arango P., Kuhlthau K.A., Simmons J.M., Neff J.M. (2012). Family-centered care: Current applications and future directions in pediatric health care. Matern. Child. Health J..

[85] Jolley J., Shields L. (2009). The evolution of family-centred care. J. Pediatr. Nurs..

[86] Carmen S., Teal S., Guzzetta C.E. (2008). Development, testing, and national evaluation of a pediatric patient-family–centered care benchmarking survey. Holist. Nurs. Pract..

[87] Almasri N.A., An M., Palisano R.J. (2018). Parents’ perception of receiving family-centered care for their children with physical disabilities: A meta-analysis. Phys. Occup. Ther. Pediatr..

[88] Shields L. (2015). What is “family-centred care”?. Eur. J. Pers. Cent. Healthc..

[89] Kuhlthau K.A., Bloom S., Van Cleave J., Knapp A.A., Romm D., Klatka K., Homer C.J., Newacheck P.W., Perrin J.M. (2011). Evidence for family-centered care for children with special health care needs: A systematic review. Acad. Pediatr..

[90] Perrin J.M., Romm D., Bloom S.R., Homer C.J., Kuhlthau K.A., Cooley C., Duncan P., Roberts R., Sloyer P., Wells N. (2007). A family-centered, community-based system of services for children and youth with special health care needs. Arch. Pediatr. Adolesc. Med..

[91] Salcudean A., Lica M.M. (2024). The Role of Systemic Family Psychotherapy in Glycemic Control for Children with Type 1 Diabetes. Children.

[92] Jackson A.C., Liang R.P., Frydenberg E., Higgins R.O., Murphy B.M. (2016). Parent education programmes for special health care needs children: A systematic review. J. Clin. Nurs..

[93] Cohn L.N., Pechlivanoglou P., Lee Y., Mahant S., Orkin J., Marson A., Cohen E. (2020). Health outcomes of parents of children with chronic illness: A systematic review and meta-analysis. J. Pediatr..

[94] Al-Rawashdeh S.Y., Lennie T.A., Chung M.L. (2016). Psychometrics of the Zarit burden interview in Caregivers of patients with heart failure. J. Cardiovasc. Nurs..

[95] Cousino M.K., Hazen R.A. (2013). Parenting stress among Caregivers of children with chronic illness: A systematic review. J. Pediatr. Psychol..

[96] Barker D.H., Quittner A.L. (2016). Parental depression and Pancreatic enzymes adherence in children with cystic fibrosis. Pediatrics.

[97] Fairfax A., Brehaut J., Colman I., Sikora L., Kazakova A., Chakraborty P., Potter B.K., Canadian Inherited Metabolic Diseases Research Network (2019). A systematic review of the association between coping strategies and quality of life among Caregivers of children with chronic illness and/or disability. BMC Pediatr..

[98] Kamerling S.N., Lawler L.C., Lynch M., Schwartz A.J. (2008). Family-centered care in the pediatric post anesthesia care unit: Changing practice to promote parental visitation. J. Perianesth. Nurs..

[99] Panganiban-Corales A.T., Medina M.F. (2011). Family resources study: Part 1: Family resources, family function and caregiver strain in childhood cancer. Asia Pac. Fam. Med..

[100] Trandel E.T., Pilewski J.M., Dellon E.P., Moreines L.T., Yabes J.G., Jeong K., Arnold R.M., Kavalieratos D. (2019). Symptom burden and unmet existential needs in adults with cystic fibrosis. West J. Nurs. Res..

[101] Ginsburg D.K., Salinas D.B., Cosanella T.M., Wee C.P., Saeed M.M., Keens T.G., Gold J.I. (2023). High rates of anxiety detected in mothers of children with inconclusive cystic fibrosis screening results. J. Cyst. Fibros..

[102] Burgess A., Wilcoxon L., Rushworth I., Meiser-Stedman R. (2021). Meta-Analysis found high rates of Post-Traumatic stress disorder and associated risk factors in parents following Paediatric medical events. Acta Paediatr..

[103] Goldbeck L., Babka C. (2001). Development and evaluation of a multi-family psychoeducational program for cystic fibrosis. Patient Educ. Couns..

[104] Besier T., Hölling H., Schlack R., West C., Goldbeck L. (2010). Impact of a family-oriented rehabilitation programme on behavioural and emotional problems in healthy siblings of chronically ill children. Child Care Health Dev..

[105] West C.A., Besier T., Borth-Bruhns T., Goldbeck L. (2009). Effectiveness of a family-oriented rehabilitation program on the quality of life of parents of chronically ill children. Klin. Padiatr..

[106] Goldbeck L., Hölling I., Schlack R., West C., Besier T. (2011). The impact of an inpatient family-oriented rehabilitation program on parent-reported psychological symptoms of chronically ill children. Klin. Padiatr..

[107] Schultz A., Barrett A., Balding E., Billingham W., Branch-Smith C., Grover Z., Yikilmaz G., Bourke C., Depiazzi J., Sander N. (2022). A pilot study of disease related education and psychotherapeutic support for unresolved grief in parents of children with CF. Sci. Rep..

[108] Shadi D., Jabraeili M., Hassankhani H., Alhani F., Bostanabad M.A. (2024). Development and validation of a supportive programme for family caregivers of children suffering from cystic fibrosis: Protocol for a sequential exploratory mixed-methods study. BMJ Open.

[109] Åslund C., Larm P., Starrin B., Nilsson K.W. (2014). The buffering effect of tangible social support on financial stress: Influence on psychological well-being and psychosomatic symptoms in a large sample of the adult general population. Int. J. Equity Health.

[110] Uchino B.N. (2004). Social Support and Physical Health: Understanding the Health Consequences of Relationships.

[111] Di Matteo M.R. (2004). Social support and patient adherence to medical treatment: A metaanalysis. Health Psychol..

[112] Gallant M.P. (2003). The influence of social support on chronic illness self-management: A review and directions for research. Health Educ Behav..

[113] Flewelling K.D., Sellers D.E., Sawicki G.S., Robinson W.M., Dill E.J. (2019). Social support is associated with fewer reported symptoms and decreased treatment burden in adults with cystic fibrosis. J. Cyst. Fibros..

[114] Jeffrey A., Andracchio L., Dvorak M., Lomas P., Smith B., Borowitz D. (2020). Virtual Peer Support for People With Cystic Fibrosis and Their Family Members: A Program Evaluation. J. Patient. Exp..

[115] Rayland A., Andrews J. (2023). From Social Network to Peer Support Network: Opportunities to Explore Mechanisms of Online Peer Support for Mental Health. JMIR Ment Health.

[116] Heisler M. (2006). Building Peer Support Programs to Manage Chronic Diseases: Seven Models for Success.

[117] Duff A.J., Latchford G.J. (2010). Motivational interviewing for adherence problems in cystic fibrosis. Pediatr. Pulmonol..

[118] Kow S., Rieger B., Morse K., Keens T., Wu S. (2024). The positive impact of journaling on adolescents with cystic fibrosis. Pediatr. Pulmonol..

[119] Downs J.A., Roberts C.M., Blackmorel A.M., Le Souëf P.N., Jenkins S.C. (2006). Benefits of an education programme on the self-management of aerosol and airway clearance treatments for children with cystic fibrosis. Chron. Respir. Dis..

[120] Irons J.Y., Petocz P., Kenny T.D., Chang A.B. (2019). Singing as an adjunct therapy for children and adults with cystic fibrosis. Cochrane Database Syst Rev..

[121] McNamara C., Johnson M., Read L., Vander Velden H., Thygeson M., Liu M., Gandrud L., McNamara J. (2016). Yoga Therapy in Children with Cystic Fibrosis Decreases Immediate Anxiety and Joint Pain. Evid.-Based Complement. Alternat. Med..

[122] Ellis A. (1997). Albert Ellis on rational emotive behavior therapy. Interview by Lata K. McGinn. Am. J. Psychother..

[123] Eseadi C. (2019). Rational-emotive behavioral intervention helped patients with cancer and their caregivers to manage psychological distress and anxiety symptoms. World J. Clin. Oncol..

[124] Liu Y., Ni X., Wang R., Liu H., Guo Z. (2022). Application of rational emotive behavior therapy in patients with colorectal cancer undergoing adjuvant chemotherapy. Int. J. Nurs. Sci..

[125] Arief Y.S., Krisnana I. (2014). The Application of Rational-Emotive Behavior Therapy to Reduce Stress among Mother with Leukemia Children. J. Ners..

[126] Moon J.R., Huh J., Song J., Kang I.S., Park S.W., Chang S.A., Yang J.H., Jun T.J., Han J.S. (2021). The effects of rational emotive behavior therapy for depressive symptoms in adults with congenital heart disease. Heart Lung..

[127] Ede M.O., Okeke C.I., Chinweuba N.H., Onah S.O., Nwakpadolu G.M. (2022). Testing the Efficacy of Family Health-Model of REBT on Family Values and Quality of Family Life Among Parents of Children with Visual Impairment. J Ration. Emot. Cogn. Behav. Ther..

[128] Abiogu G.C., Ede M.O., Amaeze F.E., Nnamani O., Agah J.J., Ogheneakoke C.E., Ugwuozor F.O., Obiyo N., Ezurike C., Nwosu N. (2020). Impact of rational emotive behavioral therapy on personal value system of students with visual impairment. Medicine.

[129] Okeke M.N., Onah B.N., Ekwealor N.E., Ekwueme S.C., Ezugwu J.O., Edeh E.N., Okeke P.M.D., Ndille R., Onwuadi C.C., Amedu A.N. (2023). Effect of a religious coping intervention of rational emotive behavior therapy on mental health of adult learners with type II diabetes. Medicine.

[130] Uwakwe S.I., Uwakwe C., Edeh N.I., Chukwu C.J., Enyi C., Irouwakwe C., Chukwuemeka-Nworu J.I., David A.O., Ezepue E.I., Aneke M.C. (2023). Efficacy of rational emotive behavior therapy for the improvement of knowledge and risk perception of hypertension among university lecturers in South East Nigeria. Medicine.

[131] Ede M.O., Okeke C.I., Obiweluozo P.E. (2022). Intervention for Treating Depression in Parents of Children with Intellectual Disability of Down’s Syndrome: A Sample of Nigerian Parents. J. Ration. Emot. Cogn. Behav. Ther..

[132] Grove A.B., Kurtz E.D., Wallace R.E., Sheerin C.M., Scott S.M. (2021). Effectiveness of a rational emotive behavior therapy (REBT)-informed group for post-9/11 Veterans with posttraumatic stress disorder (PTSD). Mil. Psychol..

[133] Frolli A., Ricci M.C., Di Carmine F., Orefice A., Saviano E., Carotenuto M. (2021). Emotional Rational Education Training Associated with Mindfulness for Managing Anxiety within Adolescents Affected by High-Functioning Autism: A Descriptive Study. Behav. Sci..

[134] Yang J., Han K.S. (2020). A rational emotive behavior therapy-based intervention for binge eating behavior management among female students: A quasi-experimental study. J. Eat. Disord..

[135] Victor-Aigbodion V., Eseadi C., Ardi Z., Sewagegn A.A., Ololo K., Abonor L.B., Aloh H.E., Falade T.A., Effanga O.A. (2023). Effectiveness of rational emotive behavior therapy in reducing depression among undergraduate medical students. Medicine.

[136] Qin N., Li J., Wu X., Zhang C., Luo Y., Dong X., Cao H., Wang S., Liu M., Xie J. (2023). Effects of rational emotive behavior therapy on alexithymia, anxiety, depression and sleep quality of older people in nursing homes: A quasi-experimental study. BMC Nurs..

[137] Hofmann S.G., Gómez A.F. (2017). Mindfulness-Based Interventions for Anxiety and Depression. Psychiatr. Clin. N. Am..

[138] Milo F., Imondi C., D’Amore C., Angelino G., Knafelz D., Bracci F., Dall’Oglio L., De Angelis P., Tabarini P. (2024). Short-term Psychodynamic Psychotherapy in Addition to Standard Medical Therapy Increases Clinical Remission in Adolescents and Young Adults with Inflammatory Bowel Disease: A Randomised Controlled Trial. J. Crohn’s Colitis.

[139] Nickel R., Ademmer K., Egle U.T. (2010). Manualized psychodynamic-interactional group therapy for the treatment of somatoform pain disorders. Bull. Menn. Clin..

[140] Söllner. W., Schüssler G. (2001). Psychodynamic therapy in chronic pain patients: A systematic review. Z. Psychosom. Med. Psychother.

[141] Hyphantis T., Guthrie E., Tomenson B., Creed F. (2009). Psychodynamic interpersonal therapy and improvement in interpersonal difficulties in people with severe irritable bowel syndrome. Pain.

